# Presence of microbiome decreases fitness and modifies phenotype in the aquatic plant *Lemna minor*

**DOI:** 10.1093/aobpla/plad026

**Published:** 2023-06-02

**Authors:** Mark Davidson Jewell, Sofia J van Moorsel, Graham Bell

**Affiliations:** Department of Biology, McGill University, 1205 ave Docteur Penfield, Montreal, Quebec H3A 1B1, Canada; Department of Geography, University of Zurich, Winterthurerstrasse 190, 8057 Zurich, Switzerland; Department of Biology, McGill University, 1205 ave Docteur Penfield, Montreal, Quebec H3A 1B1, Canada; Redpath Museum, McGill University, 859 Sherbrooke St West, Montreal, Quebec H3A 0C4, Canada

**Keywords:** Frond area, GXE, growth rate, intraspecific variation, Lemna minor, microbiome, plant phenotype, root length

## Abstract

Plants live in close association with microbial organisms that inhabit the environment in which they grow. Much recent work has aimed to characterize these plant–microbiome interactions, identifying those associations that increase growth. Although most work has focused on terrestrial plants, *Lemna minor*, a floating aquatic angiosperm, is increasingly used as a model in host–microbe interactions and many bacterial associations have been shown to play an important role in supporting plant fitness. However, the ubiquity and stability of these interactions as well as their dependence on specific abiotic environmental conditions remain unclear. Here, we assess the impact of a full *L. minor* microbiome on plant fitness and phenotype by assaying plants from eight natural sites, with and without their microbiomes, over a range of abiotic environmental conditions. We find that the microbiome systematically suppressed plant fitness, although the magnitude of this effect varied among plant genotypes and depended on the abiotic environment. Presence of the microbiome also resulted in phenotypic changes, with plants forming smaller colonies and producing smaller fronds and shorter roots. Differences in phenotype among plant genotypes were reduced when the microbiome was removed, as were genotype by environment interactions, suggesting that the microbiome plays a role in mediating the plant phenotypic response to the environment.

## Teaser

It is known that the plant microbiome influences its hosts, but that, depending on the context and the plant, the effect can either increase or decrease host performance. We found that for the small and free-floating aquatic plant *Lemna minor* , its microbiome reduced growth rate and changed the phenotype of the plant. These effects were consistent for several plant genotypes and only moderately depended on the context, specifically light and nutrient availability. Removing the microbiome homogenized plant phenotypes and influenced how the *L. minor* populations responded to the abiotic environment. This suggests that the microbiome at least, in part, mediates the plant's response to the environment.

## Introduction

Recent decades have seen considerable interest in the relationship between plants and their associated microorganisms, the plant microbiome (Bahram *et al.* 2018; Arias-Sánchez *et al.* 2019; Schmid *et al.* 2019; Tan *et al.* 2021). It is well known that a taxonomically rich assemblage of microbes (also termed ‘the second plant genome’) colonizes every accessible plant tissue, often with important consequences for plant functioning and fitness (Berendsen *et al.* 2012). Plant-associated microbiomes may confer fitness advantages to the plant host by increasing stress tolerance (Smith *et al.* 2010; Zhu *et al.* 2010; Lau and Lennon 2012; Kivlin *et al.* 2013), resistance to pathogens (Pieterse *et al.* 2014; Compant *et al.* 2019), defence against herbivory (Hubbard *et al.* 2019) and by improving nutrient uptake resulting from associations with arbuscular mycorrhizal fungi and nitrogen-fixing bacteria, whose symbiotic associations with terrestrial plants often release their hosts from severe nitrogen or phosphorus limitation (Smith and Read 2008; Mahmud *et al.* 2020). Associations with plant growth-promoting bacteria (PGPB) and other microbial organisms are of specific interest given their potential application to increase the yields of agricultural crops (Glick 2012; de Souza *et al.* 2015). However, many reported plant–microbe interactions are negative (Bever 2003; Kulmatiski *et al.* 2008; Van der Putten *et al.* 2013) and the overall effect on plant fitness is often context-dependent (Saikkonen *et al.* 1998; Berg *et al.* 2016; Brader *et al.* 2017).

Although most work on the plant microbiome focuses on terrestrial plants, there is a growing literature investigating the consequences of microbiota for floating aquatic plants (Crump and Koch 2008; Xie *et al.* 2015). Much of this work has focused on *Lemna minor*, a tiny floating aquatic plant in the family *Lemnaceae* that is increasingly used as a model system for host–microbe interactions (Zhang *et al.* 2010; Acosta *et al.* 2021). Among the smallest and fastest growing of all angiosperms, *L. minor* consists of just a single floating leaf-like frond to which a single unbranched root is attached. They are widespread and abundant, often found growing on the surface of mesotrophic and eutrophic ponds, wetlands and slow-moving rivers. Reproduction is almost exclusively asexual and vegetative with daughter fronds budding from two meristematic pouches located on the mother frond’s lower surface (Landolt 1986; Lemon and Posluszny 2000). Daughter fronds may remain attached to the mother by a stipe, a stem-like bundle of vascular tissue, resulting in colonies of varying sizes, before splitting apart after abscission severs the stipe (Landolt 1986; Lemon *et al.* 2001). In the wild, the fronds and roots are covered in a species-rich assemblage of microbes (Gilbert *et al.* 2018; Acosta *et al.* 2020), which can be removed by sterilization in the lab (Bowker *et al.* 1980). Diatoms, green algae, ciliates, rotifers and other microbes commonly associate with *L. minor* (Coler and Gunner 1969; Zuberer 1984; Madoni 1991; Goldsborough 1993). These eukaryotes are often key players in microbial food webs with the potential to alter effects of bacteria on hosts. However, host–microbiome work, in general, has focused narrowly on bacteria and fungi. Interest in the *L. minor* microbiome dates back to the early 20th century, with the observation of an association with N-fixing bacteria (Bottomley 1920), and has accelerated in recent years (Ishizawa *et al.* 2017*a*, *b*, 2019; Gilbert *et al.* 2018; Chen *et al.* 2019; Acosta *et al.* 2020; Iwashita *et al.* 2020; O’Brien *et al.* 2020*a*, *b*; Tan *et al.* 2021) with a general consensus that plant–microbe interactions play an important role in mediating plant fitness and function. For *L. minor*, almost all recent research has focused on the bacterial component of its microbiome, whose core assemblage consists of largely Proteobacteria and Actinobacteria and bears a close resemblance to the leaf microbiome in terrestrial plants like *Arabidopsis* and rice (Acosta *et al.* 2020). While certain strains of bacteria have been shown to promote growth in *L. minor*, it remains unknown how the full microbial assemblage, including eukaryotes, impacts host fitness. The small size of this plant makes it well-suited to highly replicated experiments, and its fast generation time and vegetative asexual reproduction mean that the life-time fitness can be measured across multiple generations within a single experiment as population growth rate.

The effect of the microbiome on host fitness and phenotype may depend on both the environment and the host genotype. Plant genotypes often differ in their responses to abiotic environmental conditions (Rehfeldt *et al.* 2002; Wilczek *et al.* 2014). These G × E (genotype by environment) interactions have been shown in some cases to depend on the microbiome, whose composition may vary among plant genotypes (Wagner *et al.* 2016; O’Brien *et al.* 2020*a*), or whose impact may mediate plant phenotypic responses to the environment. For example, microbially mediated shifts in plant phenotype have been shown to affect plant tolerance to environmental stress (Wagner *et al.* 2014; Hubbard *et al.* 2019; O’Brien *et al.* 2019). Furthermore, certain environmental conditions can lead to the decoupling of plant–microbe mutualisms (Shantz *et al.* 2016). Thus, the traits and fitness of the host plant may depend on the host genotype, its microbiome, the abiotic environment and the interactions between these three factors.

In this study, we have three aims. First, we ask how the presence of the *L. minor* microbiome affects plant fitness measured as growth rate. Second, we ask whether the effects of the microbiome on plant fitness are dependent on the abiotic environment and associated with changes in plant phenotypic plasticity. Third, we ask if different plant genotypes and their associated microbiomes differ in terms of the host response to the environment (G × E) and whether this is driven by the presence of the microbiome. To address these questions, we carried out a *L. minor* growth assay with both biological replication (eight different genotypes) and technical replication. We manipulated microbiome absence vs. presence and the abiotic environment (light and nutrients) in a full factorial design and assessed variation in both plant fitness and phenotype.

## Materials and Methods

### *Lemna minor* sampling, microbiome removal and reintroduction

We identified eight natural rivers, ponds and swamps supporting populations of *L. minor* located in the greater Montreal area, Canada **(see**Supporting Information—Table S1 and Fig. S1). All sites were within a 1 km proximity to agriculture, but ranged from roughly mesotrophic to eutrophic environments and also varied in terms of other characteristics, such as water body size, pH and conductivity. In late October 2019, we took samples from each site consisting of thousands of plants, which were taken back to a research greenhouse at McGill University and maintained in samples of pond water, which were simultaneously collected from each site.

To test the effect of the microbiome on plant performance, we first removed the microbiome from all fronds, and then reintroduced it to a subsample from each site. To sterilize the plants, individual fronds were thoroughly rinsed in deionized water, submerged for approximately 3 min in 10 % bleach, and then transferred to sterile high-nutrient Hoagland’s E-medium (recipe in **Supporting Information—Table S2**). After two weeks, surviving cultures were examined with microscopy, and those that appeared axenic were transferred to agar plates made with Bold’s basal medium (Stein 1973) to promote algal growth, and to agar plates made with Yeast Extract–Peptone–Dextrose growth medium (YEPD) to promote yeast and bacterial growth. After an additional two weeks of growth in Bold’s and YEPD, cultures were again examined by microscopy. This process was repeated over several weeks until axenic cultures were obtained for each site.

Once sterility was confirmed, a single frond from each site (henceforth referred to as a genotype) was used to found isogenic lines. After one month of expansion in sterile conditions, each population (one per genotype) consisting of several hundred fronds was split in two: one which remained axenic, the other to which we reintroduced its microbiome. The original samples from all sites, consisting of untreated *L. minor* fronds with their intact microbiomes growing in their natural pond water, were maintained in open 12 L containers in the greenhouse (water added occasionally to replace that lost from evaporation), and were used to reintroduce the microbiome to the experimental populations. This was done by culturing sterilized fronds for an additional two weeks (about four generations) in their natural pond water in 1.5 L culture tubs, surrounded by untreated plants from that site with intact microbiomes. We used a floating circular boom (10 cm diameter, 5 mm width) to physically isolate the target fronds (of which there were roughly 50), but submerged roots were allowed to intermingle **[**see **Supporting Information—Fig. S2****]**. This permitted reacquisition of the microbiome, which, for *L. minor* in similar experimental conditions, has been shown to begin within 24 h and to reach a stable community after 5–14 days (Acosta *et al.* 2020). This extra step of removal and reintroduction ensured that we did not have a confounding effect of sterilization-induced selection on more robust plants. Although we did not systematically characterize the microbiome, microscopy of untreated fronds and a subset of cultures post-inoculation revealed the presence of fungal hyphae, algal cells including diatoms, flagellated and ciliated protists and rotifers, in addition to prokaryotes. We acknowledge the experimental caveat that the step of reintroduction of the microbiome to the axenic plant involves artificial establishment potentially sensitive to priority effects, and as such, the reacquired microbiome is likely not entirely representative of the true native microbiome.

### Acclimation

Once two populations (with and without the microbiome) were established for each genotype, all cultures were acclimated for two weeks in a controlled common garden setting to equilibrate maternal effects and ensure an equal physiological starting point for all plants. Each population, consisting of ~150 individuals, was grown in a stoppered 500 mL Erlenmeyer flask filled with 350 mL of diluted sterile Hoagland’s E-media ([N] = 2750 µg L^−1^ and [P] = 423.5 µg L^−1^) placed in a controlled growth chamber at the McGill phytotron (165 µmol m^−2^ s^−1^ light, 20 °C, 70 % relative humidity, with a 14/10 light–dark cycle).

### Growth assay

The main experiment consisted of a growth assay of all 16 populations (eight genotypes, each with the microbiome either present or absent) in four distinct abiotic environments, a 2 × 2 crossed treatment of light and nutrient concentration. Each assay was replicated in three flasks. This fully factorial design results in a total of 192 assays (8 genotypes × 2 microbiome treatments × 4 environment treatments × 3 replicates). Ten random individual fronds were used to inoculate each 500 mL Erlenmeyer flask filled with 350 mL of sterile Hoagland’s E-media, modified to obtain the desired treatment levels, either low nutrients ([N] = 500 µg L^−1^, [P] = 77 µg L^−1^), or high nutrients ([N] = 5000 µg L^−1^, [P] = 770 µg L^−1^). These treatment levels represent mesotrophic and eutrophic conditions. The flasks were then placed in four growth chambers in the McGill phytotron (20 °C, 70 % RH, 14/10 light–dark cycle), two of which were set at low-light conditions (30 µmol m^−2^ s^−1^) the other two at high-light conditions (300 µmol m^−2^ s^−1^). The initial common garden conditions represent intermediate light and nutrient conditions in relation to the low and high treatments in the experimental growth assay. Flasks were plugged with foam stoppers and all transfers were done using sterile techniques. The 48 flasks in each growth chamber were randomly positioned, leaving a 15 cm boundary from the chamber wall.

The growth assay lasted for a total duration of four weeks after which population growth rates and plant phenotypes were measured. Four weeks translated to roughly three generations in the slowest growing environment, which is sufficient time in treatment conditions to minimize maternal and carryover effects from the acclimation phase. However, to maintain populations in a state of exponential growth, the growth assay was broken into two two-week assays. After the first two weeks of growth in the treatment environments, before fronds reached complete surface cover (on average ~100 fronds per flask), 10 randomly sampled fronds from each flask were transferred to an identical treatment flask of fresh media. The flasks were repositioned randomly in the growth chambers following the mid-assay transfer.

At the end of the experiment, the total number of fronds and the number of colonies (groups of attached fronds) were recorded for each flask. Population growth rates were measured only over the final 2-week period of the assay to remove any lag in physiological acclimation. From each flask, we randomly sampled 10 individuals (on average ~10 % of the population) on which we measured frond area and root length by imaging (plants were pressed onto a plastic sheet and photographed from a standard 20 cm distance) and subsequent image analysis using ImageJ (Abràmoff *et al.* 2004). Only mature individuals (those from which a daughter frond was budding) were included.

### Statistical analysis

The experiment had five response variables: population growth rate (fitness), colony size (number of attached fronds), frond area, root length, and shoot-to-root ratio (ratio between frond area in mm^2^ and root length in mm). Growth rate was calculated for each flask during the final two-week growth period using the standard formula for exponential growth $r=ln\left(\frac{N_{t}}{N_{0}}\right)/t$, where *N*_0_ is initial population size, *t* is time in days, and *N*_*t*_ is population size at time *t*, and number of generations, *n*, was calculated as $n=ln\left(\frac{N_{t}}{N_{0}}\right)÷ln\left(2\right).$

For each response variable, we used a linear mixed-model ANOVA from the package nlme (Pinheiro *et al.* 2019) to test the effects of the microbiome and environmental conditions (two fixed factors), and genotype (one random factor). Expected Mean Squares and estimates of *F* were evaluated for fixed and random factors as described by Sokal and Rohlf (1981). In short, expected mean squares of fixed effects are the mean squares of the interactions between the fixed effect and the random effect (genotype) instead of the residual mean square. Since there is only a single measure of growth rate per flask, replicate flask was used as the error variance, and similarly, trait values were averaged across the 10 sampled individuals and analysed at the level of the flask. Mixed models were compared to simple three-way models including genotype as a fixed factor. To assess whether the presence of the microbiome mediates phenotypic responses via an effect on fitness, we did ANCOVA using the linear model function (lm) in R for each trait (frond area, root length, shoot:root and colony size) with the presence/absence of the microbiome as the fixed factor and fitness as a covariate. All statistical analyses were done in R Version 1.0.153 (R Development Core Team 2022).

## Results

### Impact of abiotic treatments on growth rate (fitness) and phenotype

**Figure 1.**
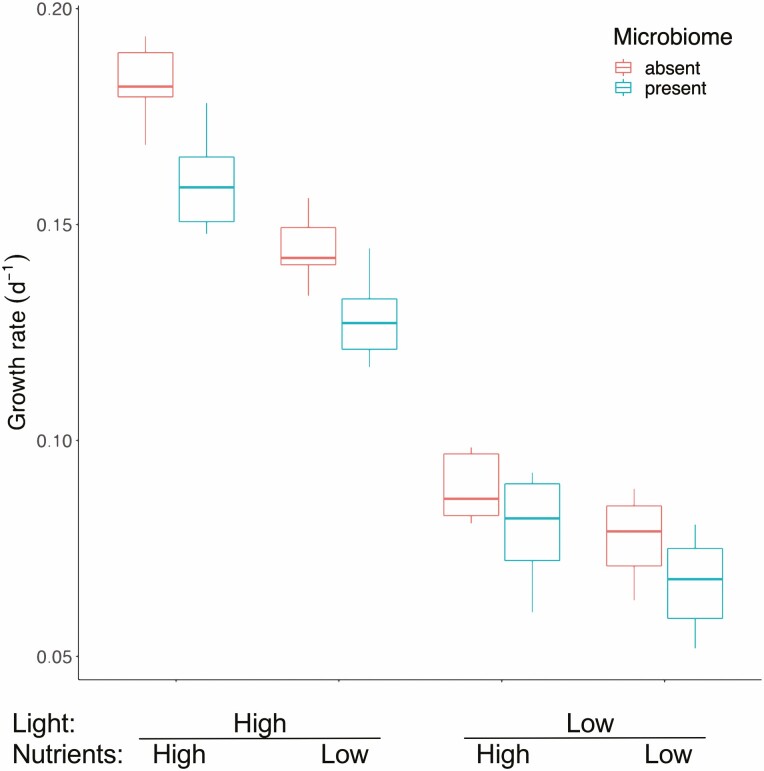
Population growth rate (fitness) for *Lemna minor* assayed in four modified abiotic conditions, with and without its natural microbiome. Each box and whisker represent the variation among eight genotypes (three replicate flasks were averaged for each of the eight genotypes). Boxes represent the upper and lower quartiles and whiskers represent maximum and minimum values.

**Figure 2.**
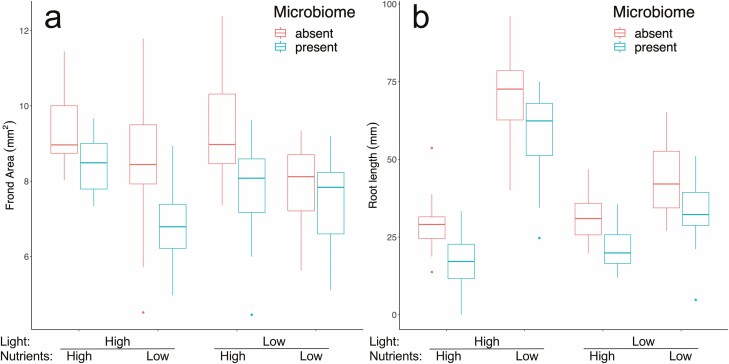
Phenotypic consequences of growth in four modified environmental conditions for *Lemna minor*, with and without its microbiome. Each box and whisker show the variation among eight independent populations (three replicate flasks were averaged for each of the eight genotypes). Boxes represent the upper and lower quartiles, whiskers represent maximum and minimum values, and outliers are shown as points. (A) Variation in frond area (mm^2^) and (B) variation in root length (mm).

**Figure 4.**
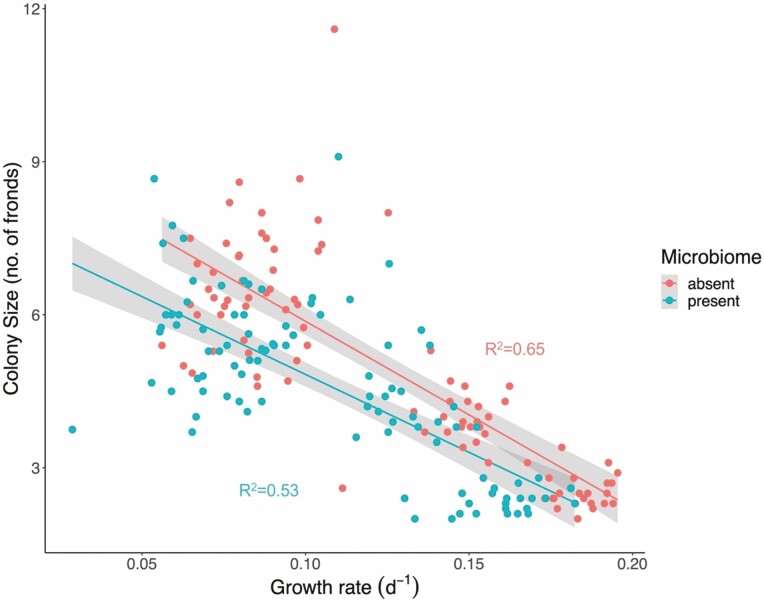
*Lemna minor* colony size (number of attached fronds in a growth unit) as a function of growth rate for populations with and without their microbiomes. Each regression is the result of 96 points (8 genotypes × 4 environments × 3 replicates). Shading around each regression line are 95% confidence intervals. See **Supporting Information—Fig. S4** for corresponding plots for frond area and root length.

The plants grew rapidly over the growth assay with an average doubling time of 6 days across all treatments. Plants grew 1.23× faster in high-nutrient conditions than in low-nutrient conditions, and more than 1.95× faster in high-light conditions than in low light (*F*_3, 21_ = 362.6, *P* < 0.001, Fig. 1). In the most favourable conditions (high light–high nutrients), average doubling time was 4 days, whereas in the most stressful conditions (low light–low nutrients), it was 10 days. Fronds grown in low-light conditions were visibly darker green in colour than those grown in high light **[**see **Supporting Information—Fig. S3****]**. Frond area was on average 1.1× times larger when grown in high-nutrient conditions compared to low-nutrient conditions (*F*_3, 21_ = 21.20, *P* < 0.001, Fig. 2A). Root length increased by 2.1× in low-nutrient conditions, although this response was stronger in high-light conditions (*F*_3, 21_ = 80.09, *P* < 0.001, Fig. 2B). Average colony size, i.e. frond aggregation, was smaller in high-nutrient and light conditions and larger when resources were low (*F*_3, 128_ = 162.49, *P* < 0.001) meaning that the slower growing the population, the greater the number of fronds that remained attached (*F*_3, 188_ = 96.7, *P* < 0.001, *m* = −36.7, *R*^2^ = 0.60, Fig. 4).

### Impact of the microbiome on plant growth and phenotype

**Figure 3.**
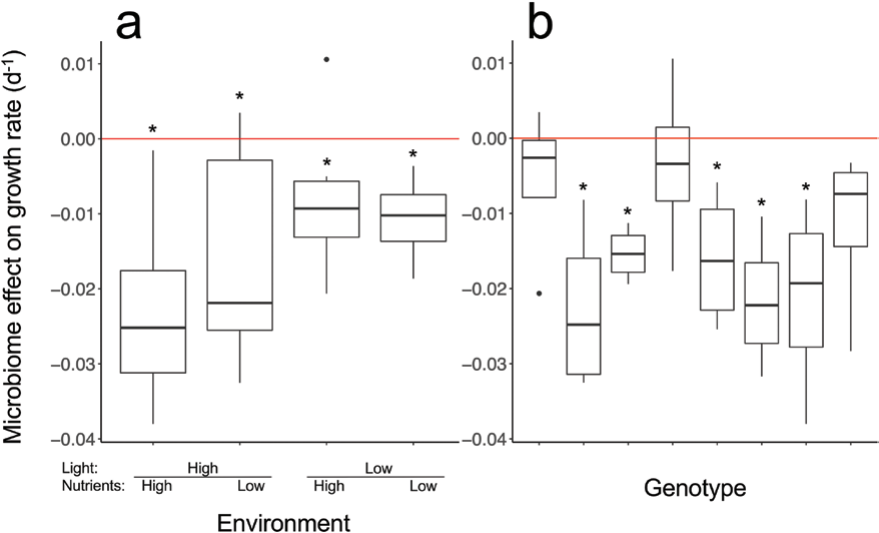
Change in growth rate attributed to the presence of the microbiome measured as the difference in mean growth rate between the two microbiome treatments (present or absent). The red zero line represents no effect of the microbiome on growth rate. Boxes represent the upper and lower quartiles and whiskers represent maximum and minimum values. Outliers are shown as points. (A) Variation in the microbiome effect among assay environmental conditions. All eight genotypes are grouped within each set of treatment conditions. (B) Variation in the microbiome effect among plant genotypes. All four levels of environment are grouped for each of eight genotypes. Significant differences in growth rates between microbiome treatments (using the Bonferroni correction) are indicated with a *.

Presence of the microbiome had a strong and consistent negative effect on plant growth rate in all environment treatments (*F*_1, 7_ = 32.3, *P* < 0.001), reducing growth rate in all treatments (Figs. 1 and 3A). Mean growth rate across all environments was lower with the microbiome present for all eight genotypes (Fig. 3B), five of which were significant using the conservative Bonferroni correction. The magnitude of this negative effect therefore varied among genotypes as well as among environmental conditions leading to significant interactions between microbiome × genotype (*F*_7, 128_ = 3.39, *P* = 0.002), and microbiome × environment (*F*_3, 128_ = 5.63, *P* = 0.001). The presence of the microbiome resulted in systematically smaller fronds (*F*_1, 128_ = 50.05, *P* < 0.001) across all environmental treatments (Fig. 2A), and for all genotypes. The extent of this, however, varied among environments and among genotypes resulting again in significant microbiome × environment (*F*_3, 128_ = 5.72, *P* = 0.001) and microbiome × genotype interactions (*F*_7, 128_ = 2.09, *P* < 0.049). Furthermore, the G × E interaction was mediated by the microbiome resulting in a significant three-way interaction (*F*_21, 128_ = 2.19, *P* = 0.004). Full ANOVA tables for all analyses with genotype as random factor can be found in Table 1. Colony size also depended on the presence of the microbiome. The presence of the microbiome decreased colony size in all environments (*F*_1, 23_ = 7.52, *P* = 0.011). Although the slope of the trait–fitness relationship between colony size and growth rate was the same whether the microbiome was present or absent (*F*_1, 188_ = 2.27, *P* = 0.13), the intercept was significantly different (*F*_1, 188_ = 7.09, *P* = 0.008), such that for the same growth rate, populations of plants with their intact microbiomes had smaller colonies across all measured growth rates (Fig. 4). We saw similar effects for frond area and root length, with both traits being modified by the presence vs. absence of the microbiome independently of growth rate **[**see **Supporting Information—Fig. S4****]**.

**Table 1.** Mixed-model ANOVA results for five response variables: A, fitness; B, frond area; C, root length; D, colony size and E, shoot:root. Microbiome and environment were evaluated as fixed factors, and genotype as a random factor.

Factors	DF	Sum Sq	Mean Sq	*F*	*P*
A. Fitness (*r*/day)					
Fixed terms					
Microbiome	1	0.0103	0.0103	32.3	<0.001
Environment	3	0.3047	0.1016	362.6	<0.001
Mic × Env	3	0.0016	0.0005	5.63	0.001
Random terms					
Genotype	7	0.0045	0.0006	6.74	<0.001
Mic × Gt	7	0.0022	0.0003	3.39	0.002
Env × Gt	21	0.0058	0.0003	2.92	<0.001
Mic × Env × Gt	21	0.0023	0.0001	1.15	ns
Residual	128	0.0121	0.0001		
Total	191	0.3465			
B. Frond area (mm^2^)					
Fixed terms					
Microbiome	1	71.07	71.07	50.05	<0.001
Environment	3	54.69	18.23	21.20	<0.001
Mic × Env	3	11.65	3.88	5.72	0.001
Random terms					
Genotype	7	92.40	13.20	19.44	<0.001
Mic × Gt	7	9.94	1.42	2.09	0.049
Env × Gt	21	18.15	0.86	1.27	ns
Mic × Env × Gt	21	31.24	1.49	2.19	0.004
Residual	128	86.92	0.68		
Total	191	376.06			
C. Root length (mm)					
Fixed terms					
Microbiome	1	5665	5665	52.45	<0.001
Environment	3	50 453	16 818	80.09	<0.001
Mic × Env	3	41	14	<1	ns
Random terms					
Genotype	7	5113	730	12.06	<0.001
Mic × Gt	7	755	108	1.78	ns
Env × Gt	21	4408	210	3.47	<0.001
Mic × Env × Gt	21	1996	95	1.57	ns
Residual	128	7750	61		
Total	191	76 181			
D. Colony size (no. of aggregated fronds)					
Fixed terms					
Microbiome	1	9.7	9.70	11.02	0.013
Environment	3	404.6	134.87	162.49	<0.001
Mic × Env	3	12.7	4.24	4.07	0.008
Random terms					
Genotype	7	25.4	3.63	3.49	0.002
Mic × Gt	7	6.2	0.88	<1	ns
Env × Gt	21	17.5	0.83	<1	ns
Mic × Env × Gt	21	44.7	2.13	2.05	0.008
Residual	128	133.3	1.04		
Total	191	654.1			
E. Shoot-to-root ratio					
Fixed terms					
Microbiome	1	0.21	0.209	8.038	0.025
Environment	3	3.97	1.323	41.344	<0.001
Mic × Env	3	0.28	0.093	7.75	<0.001
Random terms					
Genotype	7	0.41	0.059	4.917	<0.001
Mic × Gt	7	0.18	0.026	2.167	0.041
Env × Gt	21	0.67	0.032	2.667	<0.001
Mic × Env × Gt	21	0.60	0.028	2.333	0.002
Residual	128	1.54	0.012		
Total	191	7.86			

### Variation among genotypes and G × E interactions

To test the variation among genotypes, we did an additional three-way ANOVA with the same response variables but with genotype as a fixed factor **[see Supporting Information—Table S3****]**. There was considerable variation in growth rate among genotypes (*F*_7, 128_ = 6.74, *P* < 0.001); however, all eight genotypes responded to light and nutrients in the same direction (increased growth rate with higher resource levels). The extent of this increase varied among genotypes, leading to a significant genotype × environment interaction (*F*_21, 128_ = 2.92, *P* < 0.001). This variation among genotypes in response to the environment was consistent across both microbiome treatments (*F*_21, 128_ = 1.15, *P* = 0.31) suggesting that this variation in growth was at least in part due to genetic differences among host genotypes and not driven by their associated microbial communities. There was considerable variation in root length among genotypes (*F*_7, 128_ = 12.06, *P* < 0.001), which interacted with environmental condition (*F*_21, 128_ = 3.47, *P* < 0.001), indicating that this plastic response in phenotype to the abiotic environment differed among genotypes (G × E). Systematically across all environmental conditions, roots were shorter when the microbiome was present (*F*_1, 7_ = 52.45, *P* < 0.001), (Fig. 2C). There was again significant variation in frond area among genotypes (*F*_7, 128_ = 19.44, *P* < 0.001) although these all responded similarly to the environment (no genotype × environment interaction) (*F*_21, 128_ = 1.27, *P* > 0.05). Significance of both microbiome and environment treatments were the same for models that include genotype as a random (Table 1) or fixed term **[**see **Supporting Information—Table S3****]**.

## Discussion

### Presence of the microbiome consistently decreased growth rates and modified phenotypes of the host plants

Although considerable recent work has investigated the importance of the bacterial microbiome for *L. minor* performance (Ishizawa *et al.* 2017*a*, *b*, 2019; Gilbert *et al.* 2018; Chen *et al.* 2019; Acosta *et al.* 2020; Iwashita *et al.* 2020; O’Brien *et al.* 2020*a*, *b*, 2022; Tan *et al.* 2021), no studies to our knowledge have attempted to include the full microbiome including eukaryotes. Here, we isolate plants from eight different natural sites and assess the impact of their natural microbiomes on host fitness and phenotype over a range of abiotic conditions.

Contrary to our expectation, the presence of the microbiome consistently decreased plant fitness, on average by 12 %. This was the case across all environmental conditions (Fig. 1), and for most, but not all genotypes (Fig. 3B). Although several important plant–bacteria and plant–fungi mutualisms have been identified for *L. minor* (Acosta *et al.* 2020; O’Brien *et al.* 2020*a*, *b*; Tan *et al.* 2021), our results highlight the importance of pathogens, parasites, competitors and herbivores in the microbial assemblage. Given the discrepancy between our results and most other similar studies isolating the bacterial microbiome, these negative interactions are likely driven by the eukaryotic component of *L. minor*’s microbiome. Our results are also in line with literature documenting the importance of fungal and bacterial pathogens (Rejmankova *et al.* 1986; Underwood and Baker 1991; Zhang *et al.* 2010; Ishizawa *et al.* 2017*a*, *b*) and algal competition (van Moorsel 2022) on *L. minor* growth. In land plants, assemblages of PGPB are often unstable in the field (Parnell *et al.* 2016), and in *L. minor*, the effects of fitness-enhancing strains can be lost with the inclusion of additional strains due to non-additive effects (Ishizawa *et al.* 2017*b*).

The effects of the microbiome on plant phenotype were equally clear. The presence of the microbiome resulted in plants with shorter roots and smaller fronds across all environments (Fig. 2). One possible explanation for this decrease in frond size is the presence of photosynthetic algae that decrease nutrient availability through direct competition with *L. minor*. However, if this were the main mechanism through which the microbiome modified *L. minor* phenotype, then it would result in increased root length, a ubiquitous plastic response to decreased nutrient availability. However, we found the opposite, i.e. shorter roots when the microbiome was present (Fig. 2B), as well as an increase in the shoot-to-root ratio measured as frond area:root length (Table 1). In addition, although decreased nutrient availability generally resulted in an increase in colony size, we found that the presence of the microbiome increased frond abscission resulting in smaller colonies (Fig. 4). This is consistent with other work that has found microbially mediated shifts in colony size in *L. minor* (O’Brien *et al.* 2020*a*). We therefore, conclude that the mechanism by which the microbiome suppressed plant fitness in our experiment goes beyond resource competition. Frond abscission in response to stress has been extensively studied in *L. minor* in the ecotoxicology literature and it is well known that metal toxicity decreases colony size (Severi 2001; Li and Xiong 2004a,b; Henke *et al.* 2011; Topp *et al.* 2011; O’Brien *et al.* 2020*a*). The decrease in colony size we observe when the microbiome was present could be due to a similar phenomenon, resulting from toxic microbial secondary metabolites.

One reason for the apparent inconsistency of our results with studies that report a fitness-enhancing effect of many microbes (O’Brien *et al.* 2020*a*, *b*; Tan *et al.* 2021) is that most of these studies limit the microbial assemblage to bacteria (that can be cultivated on yeast mannitol), and exclude many important and ubiquitous microbes such as diatoms, filamentous chlorophytes and parasitic fungi (Rejmankova *et al.* 1986; Desianti 2012; Kohout *et al.* 2012; Moora *et al.* 2016). Furthermore, many studies are specifically seeking to identify only mutualistic associations with PGPB (Gilbert *et al.* 2018; Chen *et al.* 2019). Due to *L. minor*’s rapid growth rate, research is often in the context of the plant’s many industrial applications, which include biomass production as biofuel (Verma and Suthar 2015) or animal feed (Islam *et al.* 2004; Cheng and Stomp 2009). For such applications, there is an incentive to further enhance growth rate. Just as there is an effort to identify and select the most productive genetic strains of *L. minor* (Bergmann *et al.* 2000), much of the work on the *L. minor* microbiome has aimed to identify and isolate specific strains of PGPB (Yamaga *et al.* 2010; Tang *et al.* 2015; Appenroth *et al.* 2016) to increase plant growth. This bias in the literature could lead to a general impression that the microbiome is dominated by mutualistic fitness-enhancing associations. Here, we attempt to test the impact of a more complete natural microbial assemblage including fungi and protists in addition to prokaryotes. There are few studies that have tested the effect of the entire *L. minor* microbiome on plant fitness instead of just a small subset of bacteria, and those that did found conflicting results. The study that most resembles ours in design reinoculated the full bacterial community to axenic *L. minor* and found that the microbiome increased frond senescence while having no effect on growth (Underwood and Baker 1991).

A limitation of this study is the fact that we did not characterize the microbial community pre- and post-inoculation and thus, we can only speculate on the mechanisms responsible for our results. Furthermore, it is likely that microbial communities experienced species turnover during the course of the experiment. Yet, the phenotypic and fitness consequences of the microbiome were surprisingly consistent across all genotypes. This is notable since our genotype treatment included not just different plant clones, but also microbiomes from each site. Despite the possibility of strong differences in microbial community composition among genotypes, their overall effect on each plant genotype trended in the same direction. This is consistent with work that has shown the absence of plant–microbe specialization among genotypes in *L. minor* by manipulating plant genotype and microbial community source independently (O’Brien *et al.* 2020*a*) and a long-term experiment with terrestrial plants that showed little variability in associated bacterial community structure among plants of the same species, despite large differences among plant species (Schmid *et al.* 2019). It is thus conceivable that in our experiment, the eight independent microbial communities were of similar composition, at least in terms of broad functional groups and their interactions with the plant host. We also acknowledge the possibility that cross-contamination between microbiomes from different sites may explain some of the similarity in direction of microbiome effects on plants. Pathogenic microorganisms could have dispersed via air between the open-top containers and homogenized the microbial communities between the containers.

### Light and nutrient treatments had strong effects on plant growth and phenotype

Low-nutrient conditions resulted in an increase in root length. Although nutrient uptake in *L. minor* takes place via both the roots and fronds, the contribution to whole plant uptake shifts towards roots when nutrients are limited due to the increased surface area resulting from a disproportionate increase in root length (Cedergreen and Madsen 2002, 2004). This plastic response in root length was strengthened in high-light conditions, perhaps since the increase in plant growth resulted in more severe nutrient limitation. This response was combined with a decrease in frond area, as plants invested a larger portion of their biomass to root tissue (Fig. 2). The production of smaller fronds is a common response to stress in *L. minor* (Mohan and Hosetti 1999; Naumann *et al.* 2007; O’Brien *et al.* 2020*a*). In low-light conditions, fronds were also visibly darker green in colour, another standard plant response to light limitation due to an increase in leaf chlorophyll content ([**see Supporting Information—Fig. S3**]; Björkman 1981; Minotta and Pinzauti 1996).

Higher resource levels resulted in increased growth and led to increased abscission and therefore fewer fronds per colony. One possible interpretation is that in low-resource environments, daughter fronds act as a sink by continuing to receive fixed carbon from the rest of the colony through prolonged attachment. Alternatively, increased abscission in high-growth environments could be a mechanism to reduce competition in response to overcrowding (Kufel *et al.* 2018).

### The magnitude but not direction of the microbiome effect depended on the abiotic environment

The abiotic environment also mediated the effect of the microbiome. Although the microbiome generally influenced plant fitness and phenotype consistently across environments, the magnitude of these effects varied considerably. The microbiome suppressed plant fitness most substantially in high-resource environments (Fig. 3A) such that the presence of the microbiome decreased the magnitude of environmentally induced variation in fitness. Likewise, the effect of the microbiome on frond area, shoot-to-root ratio and colony size varied among environments. This is largely due to the high-light, low-nutrient treatment, in which the effect of the microbiome on plant phenotype was inconsistent with the other three environments. In this environment, the microbiome resulted in the largest reduction in frond area and, unlike in other conditions, decreased frond abscission increasing colony size. This suggests microbial species turnover among abiotic environment treatments and is supported by other work that showed an environmental dependence of the effect of the microbiome on *L. minor* (O’Brien *et al.* 2022).

### Evidence for genetic variation in traits and fitness

The plastic effects of the environment on plant phenotype and fitness varied among plants isolated from eight different sites. Although we cannot be sure that samples taken from different sites represent different genotypes, studies on natural populations of *L. minor* have shown considerable among-site genotype diversity at similar geographical scales to ours (Vasseur *et al.* 1993; Cole and Voskuil 1996; Xue *et al.* 2012), and it is reasonable to assume that samples taken from different sites represent different genotypes (Vámos *et al.* 2023). By removing environmental variation through common garden growth assays, we can estimate the variation in fitness due to genetic differences. We found small but significant differences in fitness and phenotype among genotypes, indicating some genetic control of these traits. This is consistent with previous work that finds large differences in fitness among clones (Ziegler *et al.* 2015). We also detected small genotype by environment (G × E) interactions for both fitness and phenotype in the absence of the microbiome. Although plants from all sites responded in the same direction to light and nutrients, the magnitude of these responses differed among genotypes indicating the presence of variation in the genetic control of phenotypic plasticity. Since we did not measure the abiotic environment at the original sites, we cannot link potential evolutionary adaptation of plants or their microbiomes to resource levels under the conditions of the experiment. Both the main effect of genotype (on fitness and frond area) and the genotype by environment interaction (on frond area and colony size) increased in magnitude when the microbiome was present. This suggests that in addition to genetic differences among plants, microbiomes likely differed as well, and mediated the plastic responses in phenotypic and fitness of the host plants.

### Conclusions

We conclude that the *L. minor* microbiome suppresses fitness of the host plant. This was the case for all environment treatments and for at least five of the eight genotypes. The strength of this negative effect was, however, dependent on the abiotic environment, with the microbiome suppressing plant fitness most in high-resource environments, suggesting microbial species turnover among environments. With the microbiome present, plants also modified their phenotype, producing smaller fronds and shorter roots. Plant genotypes differed in fitness and phenotype and responded differently to the abiotic environment. These G and G × E effects increased when the microbiome was present, indicating an important role of the microbiome in mediating plant response to the environment.

Although the *L. minor* microbiome has been shown to include important beneficial associations with many bacteria, their influence seems to be shadowed by pathogenic, parasitic and competitive interactions in our system, which includes eukaryotic elements of the microbiome.

Future work should focus on characterizing the prokaryotic and eukaryotic microbiomes and understanding how the abiotic environment mediates shifts in host–microbial associations.

## Supporting Information

The following additional information is available in the online version of this article—

**Table S1.** Overview of the eight sites from where the *Lemna minor* populations were sampled.

**Table S2.** Recipe for Hoagland’s E-Medium used in low, medium and high-nutrient treatments.

**Table S3.** Three-way fixed factor ANOVAs.

**Figure S1.** Top: Map of the sampling sites around Montreal, Quebec, Canada. Bottom: Photos from six of the eight sites.

**Figure S2.** Reinoculation of the microbiome back to axenic *Lemna minor* fronds.

**Figure S3.** Phenotypic consequences of growth in four modified environmental conditions for *Lemna minor*.

**Figure S4.** A) Frond area—growth rate relationship. B) Root length—growth rate. C) Shoot to root ratio—growth rate.

plad026_suppl_Supplementary_Materials

## Data Availability

Raw data from which all figures were generated are stored in the Dryad repository, DOI: 10.5061/dryad.4f4qrfjhq

## Notes

**Plants, Ecosystems and Climate. Chief Editor:** Mary Heskel
