# Supplementary material for: Presence of microbiome decreases fitness and modifies phenotype in the aquatic plant Lemna minor
Source: AoB Plants. 2023 Jun 2;15(4):plad026. doi: 10.1093/aobpla/plad026 (PMC10327544; doi:10.1093/aobpla/plad026)
Supplement: plad026_suppl_Supplementary_Materials [file plad026_suppl_supplementary_materials.docx]

**Supporting information**

**Table S1.** Overview of the eight sites from where the *Lemna minor* populations were sampled.

| Sampling date | Site name | Latitude | Longitude | Type | Conductivity | pH |
| --- | --- | --- | --- | --- | --- | --- |
| 27.10.19 | Boisbriand | 45.605142 | 73.829298 | river | 172.7 | 7.04 |
| 29.10.19 | Capricorne | 45.522952 | 73.441432 | swamp | 238.9 | 6.94 |
| 29.10.19 | St. Bruno | 45.525435 | 73.33423 | pond | 198.1 | 6.97 |
| 29.10.19 | Parc cite | 45.486928 | 73.410892 | swamp | 189 | 7.17 |
| 28.10.19 | Richelieu | 45.709192 | 73.187892 | river | 336.8 | 6.39 |
| 29.10.19 | Ecomuseum | 45.42687 | 73.935591 | pond | 284.3 | 7.08 |
| 29.10.19 | Quinn | 45.357844 | 73.924407 | pond | 335.5 | 7.01 |
| 29.10.19 | Turtle Bay | 45.468912 | 73.92244 | river | 438.1 | 6.53 |

**Table S2.** Recipe for Hoagland’s E Medium used in low, medium and high nutrient treatments. The pH was set to 5.8 before autoclaving the media.

|  | Low | Med | High |
| --- | --- | --- | --- |
| MgSO_4_ | 1.230 mg/L | 6.765 mg/L | 12.300 mg/L |
| Ca(NO_3_) x 4 H_2_O | 2.714 mg/L | 14.93 mg/L | 27.140 mg/L |
| KH_2_PO_4_ | 0.435 mg/L | 2.394 mg/L | 4.3530 mg/L |
| KNO_4_ | 1.263 mg/L | 6.944 mg/L | 12.625 mg/L |
| H_3_BO_3_ | 7.150 µg/L | 39.33 µg/L | 71.50 µg/L |
| MnCl_2_ x 4H_2_O | 4.550 µg/L | 25.03 µg/L | 45.50 µg/L |
| ZnSO_4_ x 7 H_2_O | 0.550 µg/L | 3.025 µg/L | 5.500 µg/L |
| NaMoO_4_ x 2 H_2_O | 0.225 µg/L | 1.238 µg/L | 2.250 µg/L |
| CuSO_4_ x 5 H_2_O | 0.350 µg/L | 1.925 µg/L | 3.500 µg/L |
| FeCl_3_ x 6 H_2_O | 0.048 mg/L | 0.266 mg/L | 0.484 mg/L |
| EDTA | 0.150 mg/L | 0.825 mg/L | 1.500 mg/L |

**Table S3.** Three-way fixed factor ANOVAs. The five response variables are: A. Fitness, B. Frond area, C. Root length, D. Colony size, and E. Shoot : Root.

1. FITNESS (r, day^-1^)

| Factors | DF | Sum Sq | Mean Sq | *F* | *p* |
| --- | --- | --- | --- | --- | --- |
| Microbiome | 1 | 0.0103 | 0.0103 | 109.59 | < 0.001 |
| Environment | 3 | 0.3047 | 0.1016 | 1076.07 | < 0.001 |
| Genotype | 7 | 0.0045 | 0.0006 | 6.74 | < 0.001 |
| Mic x Env | 3 | 0.0016 | 0.0005 | 5.63 | 0.001 |
| Mic x Gt | 7 | 0.0022 | 0.0003 | 3.39 | 0.002 |
| Env x Gt | 21 | 0.0058 | 0.0003 | 2.92 | < 0.001 |
| Mic x Env x Gt | 21 | 0.0023 | 0.0001 | 1.15 | ns |
| Residual | 128 | 0.0121 | 0.0001 |  |  |
| Total | 191 | 0.3465 |  |  |  |

B. FROND AREA (mm^2^)

| Factors | DF | Sum Sq | Mean Sq | *F* | *p* |
| --- | --- | --- | --- | --- | --- |
| Microbiome | 1 | 71.07 | 71.07 | 104.66 | < 0.001 |
| Environment | 3 | 54.69 | 18.23 | 26.85 | < 0.001 |
| Genotype | 7 | 92.40 | 13.20 | 19.44 | < 0.001 |
| Mic x Env | 3 | 11.65 | 3.88 | 5.72 | 0.001 |
| Mic x Gt | 7 | 9.94 | 1.42 | 2.09 | 0.049 |
| Env x Gt | 21 | 18.15 | 0.86 | 1.27 | ns |
| Mic x Env x Gt | 21 | 31.24 | 1.49 | 2.19 | 0.004 |
| Residual | 128 | 86.92 | 0.68 |  |  |
| Total | 191 | 376.06 |  |  |  |

C. ROOT LENGTH (mm)

| Factors | DF | Sum Sq | Mean Sq | *F* | *p* |
| --- | --- | --- | --- | --- | --- |
| Microbiome | 1 | 5665 | 5665 | 93.57 | < 0.001 |
| Environment | 3 | 50453 | 16818 | 277.77 | < 0.001 |
| Genotype | 7 | 5113 | 730 | 12.06 | < 0.001 |
| Mic x Env | 3 | 41 | 14 | < 1 | ns |
| Mic x Gt | 7 | 755 | 108 | 1.78 | ns |
| Env x Gt | 21 | 4408 | 210 | 3.47 | < 0.001 |
| Mic x Env x Gt | 21 | 1996 | 95 | 1.57 | ns |
| Residual | 128 | 7750 | 61 |  |  |
| Total | 191 | 76181 |  |  |  |

D. COLONY SIZE (no. of aggregated fronds)

| Factors | DF | Sum Sq | Mean Sq | *F* | *p* |
| --- | --- | --- | --- | --- | --- |
| Microbiome | 1 | 9.7 | 9.70 | 9.32 | 0.003 |
| Environment | 3 | 404.6 | 134.87 | 129.50 | < 0.001 |
| Genotype | 7 | 25.4 | 3.63 | 3.49 | 0.002 |
| Mic x Env | 3 | 12.7 | 4.24 | 4.07 | 0.008 |
| Mic x Gt | 7 | 6.2 | 0.88 | < 1 | ns |
| Env x Gt | 21 | 17.5 | 0.83 | < 1 | ns |
| Mic x Env x Gt | 21 | 44.7 | 2.13 | 2.05 | 0.008 |
| Residual | 128 | 133.3 | 1.04 |  |  |
| Total | 191 | 654.1 |  |  |  |

E. SHOOT : ROOT (on raw data)

| Factors | DF | Sum Sq | Mean Sq | *F* | *p* |
| --- | --- | --- | --- | --- | --- |
| Microbiome | 1 | 0.21 | 0.209 | 17.42 | < 0.001 |
| Environment | 3 | 3.97 | 1.323 | 110.25 | < 0.001 |
| Genotype | 7 | 0.41 | 0.059 | 4.917 | < 0.001 |
| Mic x Env | 3 | 0.28 | 0.093 | 7.75 | < 0.001 |
| Mic x Gt | 7 | 0.18 | 0.026 | 2.167 | 0.041 |
| Env x Gt | 21 | 0.67 | 0.032 | 2.667 | < 0.001 |
| Mic x Env x Gt | 21 | 0.60 | 0.028 | 2.333 | 0.002 |
| Residual | 128 | 1.54 | 0.012 |  |  |
| Total | 191 | 7.86 |  |  |  |


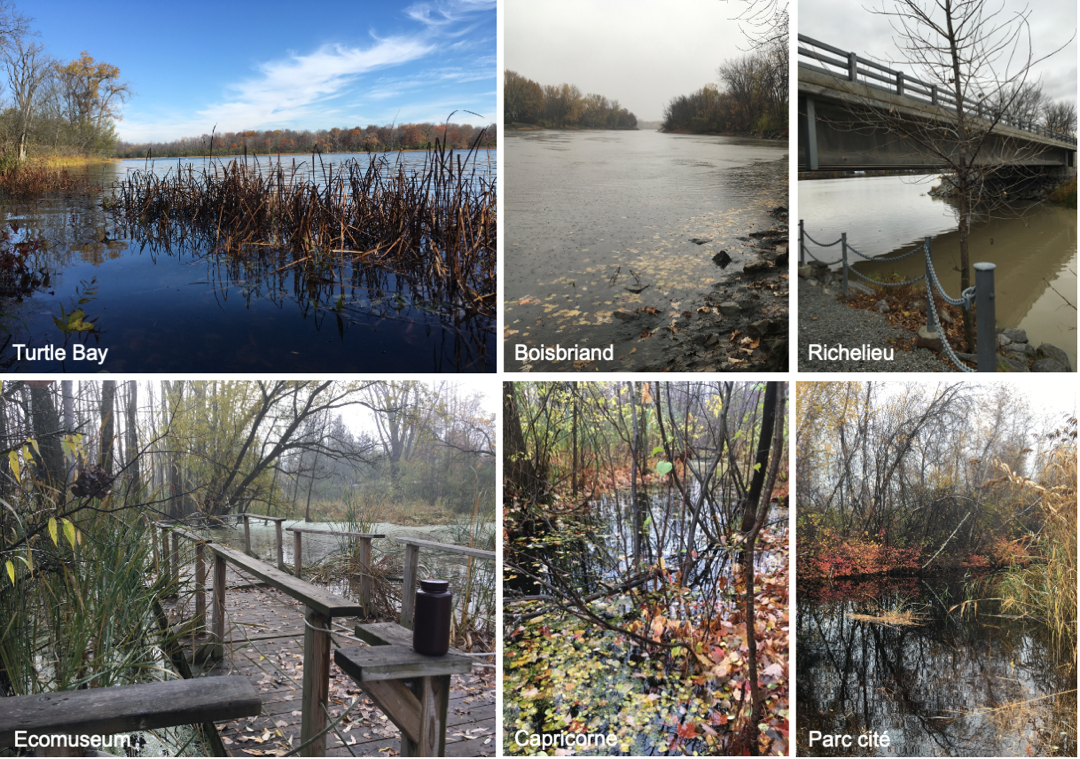

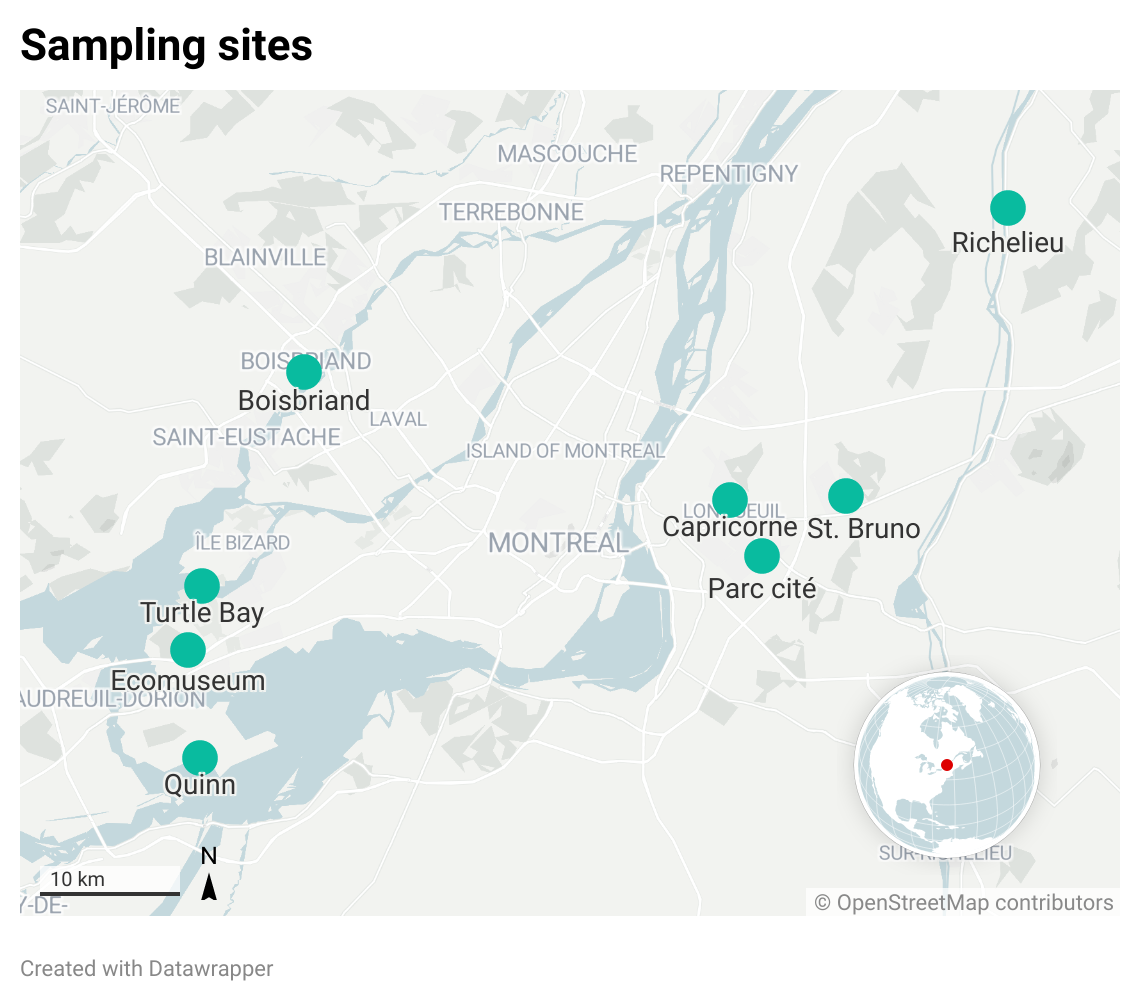


**Figure S1.** Top: Map of the sampling sites around Montreal, Quebec, Canada. Created with Datawrapper. Bottom: Photos from six of the eight sites.

*.*


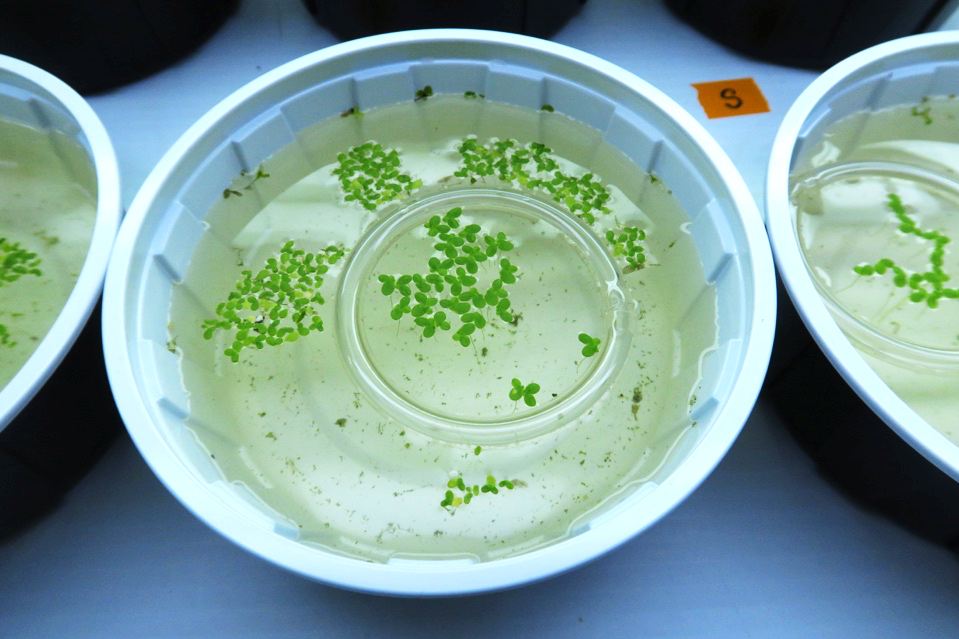


**Figure S2.** Reinoculation of the microbiome back to axenic *Lemna minor* fronds. The container, filled with natural pond water is filled with fronds from the same site, with their microbiome intact. The floating circular boom isolates the sterilised target fronds. Although the fronds are spatially separated at the water’s surface, the roots intermingle. To reinoculate the microbiome back to the axenic fronds, the target fronds were cultured here for two weeks (about two to three generations).


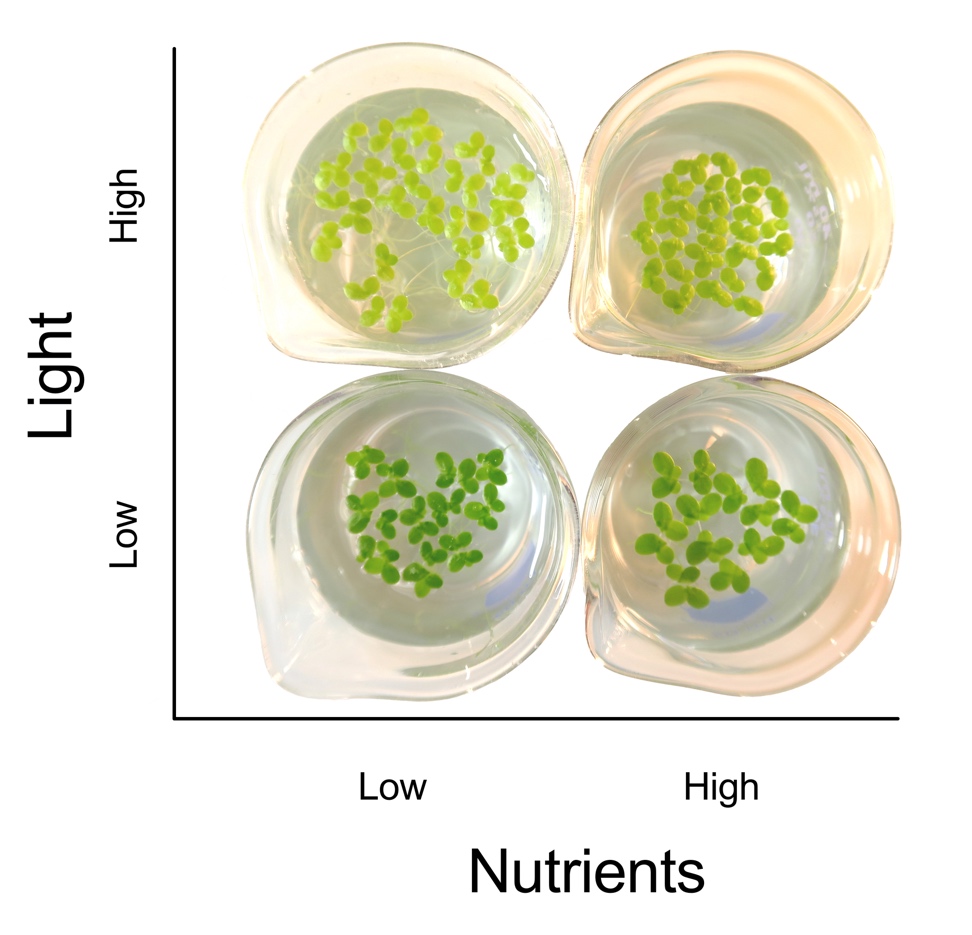


**Figure S3.** Phenotypic consequences of growth in four modified environmental conditions for *Lemna minor*. Photos are of a single genotype grown in four abiotic environments, without the microbiome.


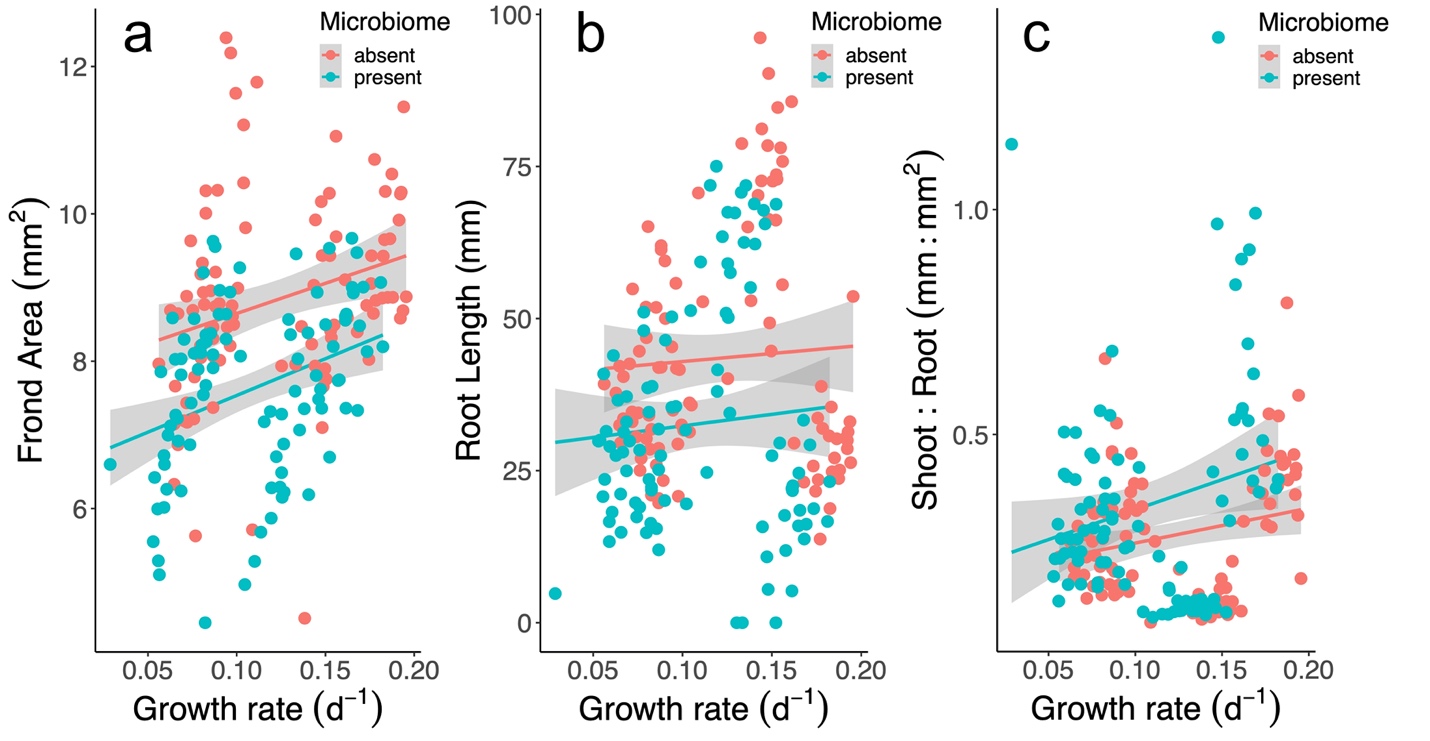


**Figure S4.** a) Frond area – growth rate relationship. B) Root length – growth rate. C) Shoot to root ratio – growth rate. For all three traits, the interaction between growth rate and microbiome presence/absence was not significant (F_1,188_=0.173, p=0.68 for frond area, F_1,188_=0.03, p=0.86 for root length, F_1,188_=0.64, p=0.43 for shoot root ratio).
